# What is the Real Clinical Significance of Carotid Sinus Hypersensitivity in Clinical Practice? A Dilemma Still Waiting for Answers

**DOI:** 10.36660/abc.20200005

**Published:** 2020-02

**Authors:** Tan Chen Wu

**Affiliations:** Hospital das Clínicas Instituto do Coração, Faculdade de Medicina da Universidade de São Paulo, SP - Brazil

**Keywords:** Carotid Sinus Massage/physiopathology, Syncope, Aged, Hypotension, Mortality, Cardiac Pacing, Artificial

Carotid sinus hypersensitivity (CSH) is defined by pause ≥ 3 seconds (sinus or atrioventricular block) and/or systolic blood pressure drop ≥ 50mmHg during carotid sinus massage (CSM).^1^ The prevalence of CSH varies according to the method and population evaluated in up to 68% of elderly patients with syncope and 35% of asymptomatic individuals over 65 years of age.^2^ Therefore, the cause-effect relationship between carotid sinus hypersensitivity and syncope should always be questioned, and may only be a casual finding and not necessarily the carotid sinus syndrome (CSS), one of the causes of syncope seen mainly in elderly patients.^3^

The clinical relevance of CSH obtained with CSM was questioned in a study recently published by Wu et al.^4^ The authors compared the response to CSM between 99 patients with syncope to clarify and 66 asymptomatic patients and found similar rates of CSH between the two groups, with cardioinhibitory response in 24.2 and 25.8% and vasodepressor response in 8.1 and 13.6%, in symptomatic and asymptomatic patients respectively (p = 0.466).^4^ Therefore, CSH may be a nonspecific response in the evaluation of syncope in these patients with dubious clinical significance, especially in the elderly population with multiple comorbidities, often with the possibility of varying etiologies.

Attempts to refine or modify the definition of positive response have been proposed to enable accuracy in the diagnosis of CSS as the cutoff value of systolic blood pressure ≤ 85 mmHg combined with symptoms suggested by Solari et al.^5^ The authors concluded that one-third of the 164 patients evaluated with isolated vasodepressor form could not be identified with the current criterion (systolic blood pressure drop ≥ 50 mmHg) compared to the cutoff value of ≤ 85 mmHg systolic blood pressure. Krediet and colleagues^6^ also questioned the current criteria for CSH, considering them to be very sensitive, resulting in the high prevalence observed in the elderly population.^6^ They suggested changing to pause ≥ 6 seconds and/or lowering mean blood pressure to < 60 mmHg for more than 6 seconds, based on the fact that 6 seconds of asystole are required to cause loss of consciousness;^7^ that in the general population the 95th percentile for response to CSM was 7.3 seconds of asystole;^6^ that in clinical follow-up, patients with pauses > 6 seconds (43%) had significant recurrence of syncope compared to patients with 3-6 seconds who had only 0.7% of occurrence;^8^ and that in the International Study on Syncope of Uncertain Etiology 2 (ISSUE-2) the average pause in observed syncope recurrence was 9 seconds (8-18 seconds).^9^ Based on this new criterion, McDonald and colleagues analyzed mortality according to the current criterion and the criteria proposed by Krediet (described above) and Kerr (pause > 7.3 seconds and systolic blood pressure drop > 77 mmHg).^10^ In a total of 272 patients, 106 of them (38.9%) had CSH according to the standard criteria, and 141 (51.8%) and 28 (10.3%) according to the Krediet’s and Kerr’s criteria.^10^ They did not observe statistical difference in mortality in patients with and without CSH in a mean follow-up of 8.6 years by the standard criterion (32*vs*. 22%, respectively p = 0.073), but noted differences according to Krediet’s (33*vs*. 19%, p = 0.009) and Kerr’s (53 *vs*. 23%, p < 0.001) criteria. After adjusting for age and gender, only CSH defined by Kerr’s criterion was associated with increased total mortality (risk rate 2.023, 95%CI 1.131-3.618, p = 0.009).

In this issue, the study by Lacerda and colleagues^11^ observed the evolution of 502 patients undergoing CSM, with 52 patients presenting cardioinhibitory response or asystole ≥ 3 seconds. When compared to the 408 patients with physiological response (or without CSH), the authors did not observe differences in either cardiovascular or trauma-related mortality, with total mortality rates of 55.8 *vs*. 49.3% (p = 0.38) in patients with and without cardioinhibitory response respectively.^11^ Among the 52 patients with cardioinhibitory response to CSM, only 7 patients had a history of syncope and no pacemaker implantation was required in any of them. The low prevalence of patients with syncope in the study, placed as a limitation, may have further reinforced the indifference in the evolution of patients with or without cardioinhibitory CSH. These results reinforce the hypothesis of the limitation of CSH findings to clinical applicability in most of the observed cases and are in agreement with the current literature.

Therefore, CSH remains a matter of evaluation, with controversy since its definition, based predominantly on small, old studies with technical limitations of the time, and the heterogeneity of the methods employed in the CSM. The lack of accuracy has been pointed as a factor in the low specificity of the finding, making it difficult, and sometimes confusing the clinician, for the proper diagnosis of CSS in the investigation of syncope to be performed, which requires response to CSM according to the criteria for CSH combined with reproduction of clinical symptoms during the maneuver. The findings of the article rekindle, once again in the literature, the need for reevaluation of the current parameters described in the consensus on CSH, the bases for the correct diagnosis, appropriate treatment and prognosis of CSS in syncope.
